# Effects of sensory combination on crispness and prediction of sensory evaluation value by Gaussian process regression

**DOI:** 10.1371/journal.pone.0297620

**Published:** 2024-02-08

**Authors:** Hiroyuki Nakamoto, Ryoga Nishimura, Futoshi Kobayashi

**Affiliations:** Graduate School of System Informatics, Kobe University, Kobe, Hyogo, Japan; University of Sharjah, UNITED ARAB EMIRATES

## Abstract

Crispness contributes to the pleasantness and enjoyment of eating foods and is popular with people of wide ages in many countries. Hence, a quantitative evaluation method for crispness is required for food companies developing new food products. In this study, the effects of different sensory combinations on crispness were investigated through sensory evaluation, and a Gaussian process regression model was used to predict the evaluation values of crispness. First, four crispness descriptors in Japanese were selected, and sensory evaluations were conducted with ten participants using commercially available snack foods under three different sensory combinations of force, vibration, and sound to confirm the effects of the three senses. An instrumental system also measured force, vibration, and sound for snack foods under the same conditions. The Gaussian process regression model determined the relationship between the sensory and measurement data and predicted the sensory evaluation values from the measurement data. Cross-validation verified that the Gaussian process regression model accurately predicted the food texture evaluation values from the measurement data even in conditions with different sensory components.

## Introduction

Crispness and crunchiness are typical textural attributes that contribute to the pleasantness and enjoyment of eating foods [1]. Crispness is used to express the texture of various kinds of food, such as fresh vegetables, baked bread, and fried snacks [2–4]. Fifty years ago, Szczesniak reported that crispness and crunchiness were the most frequently used descriptors of food texture based on a survey of 150 individuals [5]. Recently, Luckett and Seo surveyed 337 individuals in North America and asked them to pick the first three words they thought of from a large pool of words related to texture, flavor, and aroma, among others for 32 different foods [6]. They found that the most picked word from the pool of words/adjectives regarding texture was crunch/crunchy, followed by crisp/crispy. Consumers’ texture vocabulary has also been studied in other countries, including Finland, Spain, Japan, etc [7–9]. People living in non-English speaking countries use terms that are equivalent to crispness in their native language. In Austria, 208 college students were asked to describe the texture of 50 different foods using 105 texture words, and the word that appeared most frequently was crispness [10]. As crispness has long been a popular texture attribute among people of different countries and ages, the development and production of delicious and enjoyable crisp foods are important for food companies. Food companies need to evaluate crispness in order to develop foods that are acceptable to consumers.

The definitions of crispness and crunchiness have been reported in many research works. Szczesniak defined crispness as "Firm and brittle, snaps easily, emitting a typical sound upon deformation [11]." Dijksterhuis et al. developed a detailed crisp description by adding words to the crispness and then subdivided the definition of crispness [12]. In a review paper, Saeleaw and Schleining collected definitions of crispness from relative papers [2]. Most of the definitions included the expression of "sound" such as the above definition by Szczesniak. Christensen and Vickers had participants evaluate crispness by normally biting and chewing a variety of crisp foods and by biting and chewing those same foods with the sound blocked [13]. Edmister and Vickers had their participants evaluate the sounds of crisp foods by normally biting and chewing them and by only listening to the sounds of someone else eating them [14]. They evaluated the effects of sound on crispness in foods. Regarding the use of sound in crispness, Zampini and Spence reported that modulated sound changed the perceived crispness of potato chips [15]. Tunick et al. defined crispness as follows: "A dry rigid food which, when bitten with the incisors, fractures quickly, easily, and totally while emitting a relatively loud, high-pitched sound [16]." These results suggest that, in order to evaluate the crispness of food, the evaluation system should measure both force and sound data during the fracture of the food in chewing.

An instrumental method by texture profile analysis (TPA) is generally used for the texture evaluation of solid food [17]. The TPA parameters evaluate the texture attributes such as hardness and cohesiveness as physical quantities [18]. The TPA parameters are calculated from measurement data acquired by a force sensor and do not include sound information. Thus, many researchers used both force and sound measurement data to evaluate crispness [19]. Varela et al. used a load cell and a microphone to evaluate the crispness of roasted almonds based on measured force and sound [20]. Sanahuja et al. classified the crispness-related freshness of puffed snacks from force and sound data by a support vector machine, which is one of the machine learning methods [21]. de Moraes et al. analyzed the relationship between moisture and physical parameters concerning the crispness of banana snacks [22]. They reported that the numbers of force and sound peaks were related to the crispness of banana snacks. The same trend was reported in the case of potato chips [23]. Taniwaki and Kohyama indicated the magnitude of the force drop at the major fracture related to the crispness of potato chips [24]. Gouyo et al. measured French fries with a force instrument and a microphone and discussed a method to use force and sound measurement data for the evaluation of the frying process [25]. These studies indicate that the combination and force and sound is necessary for the evaluation of crispness.

Sound can be distinguished into two types, acoustic sound and vibration, which humans perceive as air-conduction sound and bone-conduction sound, respectively [26, 27]. In the studies described above, microphones measured air-conduction sound as sound. Spectra of the sound analyzed by a fast Fourier transform (FFT) had a difference between crispy and crunchy foods [27]. Neural network models classified snack foods into four grades of sensory crispness by using sound spectra [28]. On the other hand, bone-conduction sound is vibration that propagates from the teeth to the inner ear. Since the vibration occurs at the contact point or area between a tooth and food in the case of humans, an instrument should measure the vibration of a probe. Iwatani et al. developed a probe with a piezoelectric sensor and analyzed the relationship between food textures and a texture index calculated from FFT spectra [29]. Then, Sakurai et al. also developed a probe with a three-axis accelerometer and a swing-arm device [30]. Their papers reported differences in vibration measurement data occurring due to differences in food texture. Therefore, a measurement system that simultaneously measures force, vibration, and sound, and a method for evaluating texture using these measurement data, are necessary for texture evaluation.

The present study, first, confirms the effects of different sensory combinations of force, bone-conduction sound, and air-conduction sound on the sensory evaluation values of four crispness texture descriptors by sensory evaluation. Next, a measurement system obtains force, vibration, and air-conduction sound data of snack foods under the same conditions as the sensory evaluation. A Gaussian process regression, one of the machine learning methods, predicts the sensory evaluation values from the feature values extracted from the measurement data, and iterative predictions verify the prediction accuracy by cross-validation. Based on the results, the effects of sensory combinations on crispness and the potential of crispness prediction with Gaussian process regression are discussed.

## Materials and methods

### Food texture descriptors and food samples

This study chose four food texture descriptors to express crispness and crunchiness and defined them for sensory evaluation. Table 1 lists the four descriptors. Many Japanese people often use these texture descriptors that mean individually different crispness and crunchiness.

**Table 1:** Texture descriptors, their definitions, and the typical foods.

Descriptor	Definition	Typical food^†^
Sakusaku	Easily broken by biting with a weak force	Cookie, apple
Karikari	Short fracture with a relatively strong force in a mastication	Roasted nuts, unripe plum
Paripari	Breaking thin foods with a relatively high-frequency sound	Potato chips, sliced cucumber
Zakuzaku	Fractures including layers and small particles with a low-frequency sound	Shaved ice, cornflakes

^†^Extract from [9].

Eight commercially available snacks were chosen based on having crispness and occurring air-conduction sound. Table 2 lists these snack foods. Thin rice crackers (S2), potato chips (S4), and pretzel sticks (S5) are highly crispy. Sables (S1), ellipsoid rice crackers (S3), and biscuits (S6) are relatively brittle. Fried sticks of sweet potato (S7) and cubic rice crackers (S8) have crunchiness. Since they have individually different food textures regarding crispness and crunchiness, this study chose these snack foods. The sample heights are listed in Table 3.

**Table 2:** Snack samples. All samples are made in Japan.

Index	Sample	Product name, and company
S1	Sablé	Coconut sablé, Nissin Cisco Co., Ltd.,
S2	Thin rice cracker	Seven premium usuyaki senbei, Hizatsukiseika Co., Ltd.
S3	Ellipsoid rice cracker	Peanut salad, Abeko Seika Co., Ltd.
S4	Potato chip	Seven premium potato chips, Calbee Co., Ltd.
S5	Pretzel stick	Super karikari pretz, Ezaki Glico Co., Ltd.
S6	Biscuit	Macrobiha, Morinaga Co., Ltd.
S7	Fried stick of sweet potato	Imokenpi, Nangoku Seika Co., Ltd.
S8	Cubic rice cracker	Ponsuke, Mitsubishi Shokuhin Co., Ltd.

**Table 3:** Height of snack samples. Mean ± standard deviation of 10 measurements.

Index	S1	S2	S3	S4	S5	S6	S7	S8
Height mm	6.2±0.2	5.1±0.3	8.3±0.3	4.3±0.5	3.4±0.1	12.5±0.6	5.1±0.1	17.9±0.5

### Sensory evaluation

In sensory evaluation, ten panelists (eight males and two females, with an average age of 23.1 ± 0.94, mean ± standard deviation) tested the eight samples based on the quantitative descriptive analysis [31, 32]. They were recruited on October 13, 2022. Before the evaluation, the panelists were provided with Table 1 with an explanation in Japanese for each food texture. In addition, to confirm four texture descriptors, the panelists chewed four foods: pretzel sticks (Pretz, Ezaki Glico Co., Ltd., Japan), fried sticks of sweet potato (Imokenpi, Nangoku Seika Co., Ltd., Japan), rice crackers (Salada usuyaki, Ezaki Glico Co., Ltd., Japan), and Cubic granola (Frugra bits, Calbee Co., Ltd., Japan), which have mainly sakusaku, karikari, paripari, and zakuzaku textures, respectively.

To record the intensity of the four texture descriptors in Table 1, the panelists made a vertical mark at the position representing the intensity on a 120-mm horizontal line in a scorecard. The horizontal line had two-word anchors at both terminals, with the left and right terminals corresponding to "no feel" and "strong feel", respectively. After the panelists had completed their evaluations, the distance from the left endpoint to the position of the vertical mark was measured and converted into numerical data with intensity ranging from 0 to 120.

**Fig 1:**
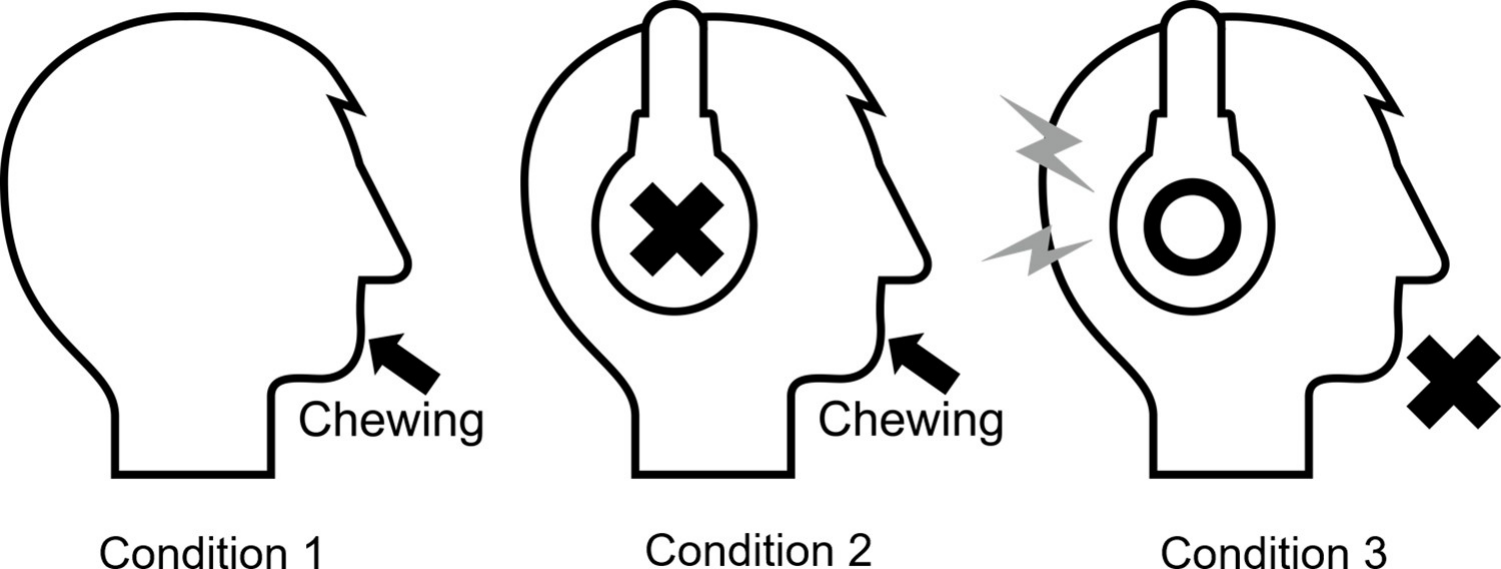
Three conditions in sensory evaluation.

The sensory evaluation included the following three conditions with different sensory combinations as shown in Fig 1.

- Condition 1 (normal): with force, bone-conduction sound, and air-conduction sound
- Condition 2 (no air sound): with force and bone-conduction sound
- Condition 3 (only air sound): with air-conduction sound

In conditions 1 and 2, the panelists took two chews of a one-bite sample, approximately 25 mm per side or length, by their molars. In condition 2, they wore earplugs (form earplugs, Moldex Co., USA) in their ears and a hearing protection ear muff (Ear muff X5A, 3M, USA) on their head. Although condition 2 completely blocked air-conduction sound, the panelists could hear bone-conduction sound. In condition 3, instead of their own chews, they heard the air-conduction sounds of two-time chew through a headphone (ATH-M20x, Audio-technica Co., Japan). These sounds were recorded with a binaural microphone (Binal 2, Wind Audio Japan Co., Japan) the sound of two-time chew generated by one author’s chews. Since the microphone has auricle parts, the author made the sound of two-time chew at the position of the mouth in relation to the auricles, 70 mm below and 70 mm in front of the auricle part.

The panelists chewed each sample twice with their molars or listened to air-conduction sound via the headphone, and then evaluated four textures by marking them on the scorecard. The sensory evaluation was performed in the order of conditions 1, 2, and 3. Each condition included the eight samples, and the order of the samples in each condition was randomized. All the panelists repeated the sensory evaluation five times for each condition. The room temperature was approximately 20°C. This study was approved by the Ethics Committee of the Kobe University Graduate School of System Informatics (No. R02-01) in accordance with the Helsinki Declaration. Written informed consent was obtained from all study participants. The sensory evaluation results were anonymized and converted into data so that individuals could not be identified. The statistical analysis of the results was carried out using MATLAB (R2022a, Mathworks, Inc, USA).

### Measurement system

**Fig 2:**
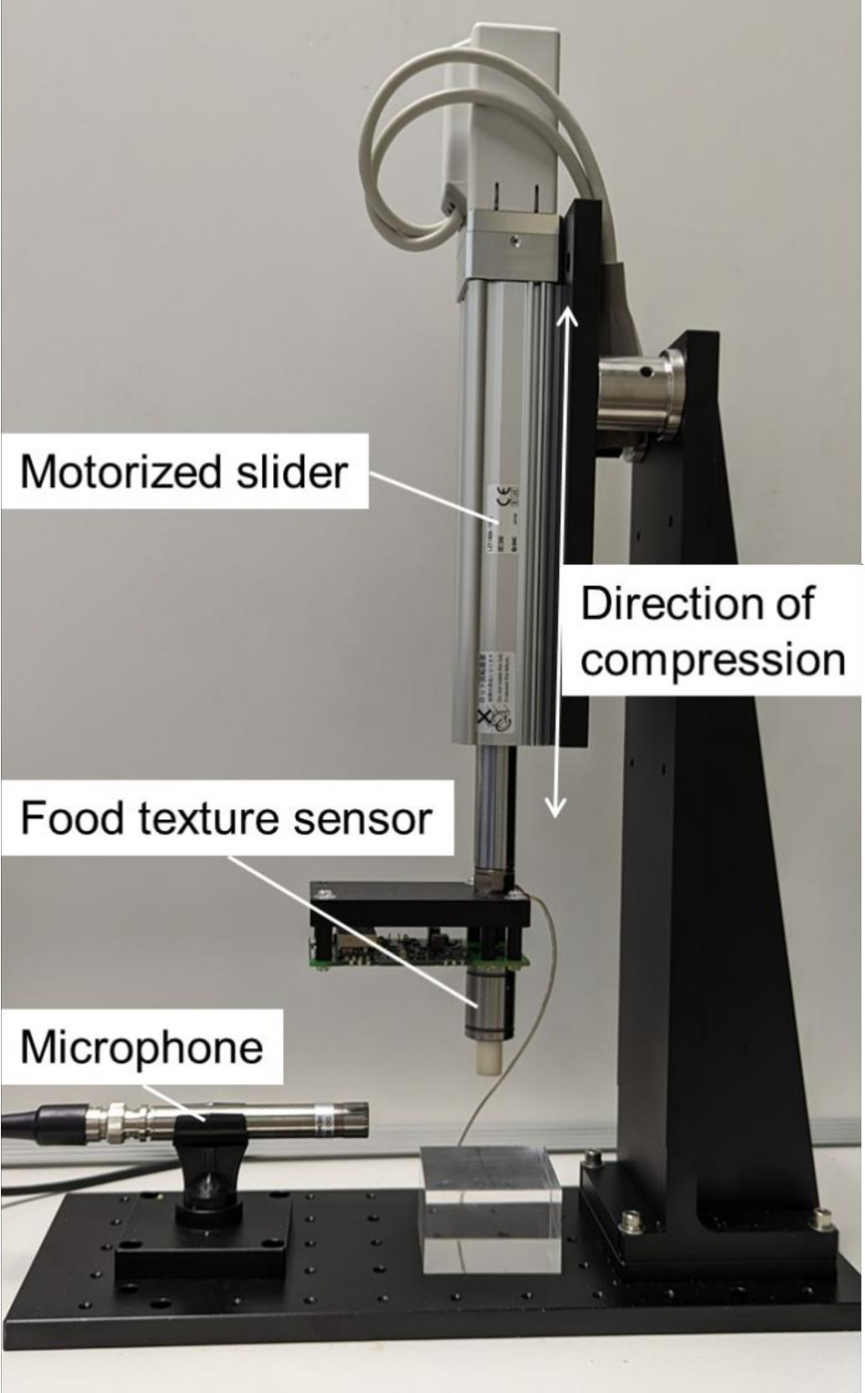
Measurement system.

The measurement system shown in Fig 2 was used to measure force, vibration (bone-conduction sound), and air-conduction sound during sample fracture. The measurement system mainly consists of a magnetic food texture sensor [33], a microphone (MI-1271M12, Ono Sokki Co. Ltd., Japan), a data recorder (DR-7100, Ono Sokki Co. Ltd., Japan), a motorized slider (LEY16DA, SMC Co. Ltd., Japan), a motor driver (LECPAN1, SMC Co. Ltd., Japan), and a desktop computer. The food texture sensor measures both force and vibration occurring on a probe, which is a part that presses a sample, with a sampling frequency of 10 kHz. The range of force is from -10 to +70 N, where + and—mean compression and tensile forces, respectively. The maximum error is within ±5% of the range. The vibration of the sensor’s probe is measured as a change of voltage due to induced electromotive force (details written in [33]). The fast vibration of the probe induces high voltage. The microphone measures sound pressure and its sampling frequency is 51.2 kHz. The probe is a cylindrical shape with a diameter of 10 mm. The distance between the probe of the texture sensor and the microphone was approximately 30 mm. The measurement system drives the motorized slider to press a sample with the probe of the texture sensor. Simultaneously, the desktop computer records the data of force, vibration, and sound.

In instrumental experiments, the measurement system pressed a sample with the texture sensor at 10-mm/s velocity from the position where it was in contact with the sample, and returned the texture sensor to its contact position. In advance of measurement, the height of each sample was measured. The distance of the movement was 80% of the height of each sample. The measurement system repeated this motion twice to simulate a two-chew motion.

### Feature values of instrumental measurement data for food texture prediction

Feature values that are used as a part of the dataset for food texture prediction were determined from the measurement data of force, vibration, and sound pressure. The feature values were calculated by Python 3.10 and MATLAB (R2022a, Mathworks, Inc, USA).

#### Force

Based on the texture profile analysis [18], six values; hardness, fracturability, adhesive force, adhesiveness, cohesiveness, and gumminess, were calculated from the force data as feature values.

#### Vibration

Firstly, the baseline of the data was set to 0. Secondly, the stationary noise of the vibration data derived from the amplifier circuit was removed. Specifically, the standard deviation of the noise was calculated from the data without vibration, and the data that did not exceed three times the standard deviation were set to 0. After taking the absolute value of the data, the time-series feature values, which consisted of the maximums of the moving average of vibration peaks during the first and second compressions, the number of vibration peaks, the mean interval of vibration peaks, mean, standard deviation, variance, skewness, and kurtosis of the vibration data were calculated. The frequency-related feature values, twelve average spectra of octave bands with center frequencies ranging from 1 to 2000 Hz, were also calculated. Thus, both the time-series and frequency-related feature values are used for the vibration data. The number of the feature values of vibration is 22.

#### Sound

The sound pressure data included a stationary noise derived from the amplifier circuit. Hence, in the same manner as the vibration data, the noise was removed. Feature values; the maximums of the moving average of pressure peaks during the first and second compressions, the number of sound pressure peaks, the mean interval of sound pressure peaks, the maximum, mean, standard deviation, variance, skewness, and kurtosis of the sound pressure were calculated from the sound data. In addition, the maximums and means of loudness and sharpness, and 23 means of a-weighted spectra in 1/3 octave bands of center frequency from 80 to 12500 Hz were calculated [34]. The number of the feature values of sound is 38.

#### Condition and dataset

The measurement system measured force, vibration, and sound to correspond to three conditions shown in Fig 1. In condition 1, the measurement system obtained all the force, vibration, and sound data. In condition 2, force and vibration, and condition 3, sound were measured, respectively. The feature values were calculated from the measurement data in three conditions. In the case of condition 2, the feature values of sound were set to 0. In condition 3, the force and vibration features were set to 0. The number of measurement replicates was 15 times per sample in each condition. Thus, the total number of the dataset was 360, 120 for each condition.

### Food texture prediction

To predict sensory evaluation values from feature values of measurement data, this study uses a Gaussian process regression (GPR) [35]. The GPR determines the relationship between the sensory evaluation values and measured feature values by a kernel function as follows:

$$k(x,x^{\prime })=\theta_{1}\exp\left(-\frac{{\left|x-x^{\prime }\right|}^{2}}{\theta_{2}}\right),$$ (1)

where ***x*** and ***x***′ are vectors of measured feature values. *θ*_1_, *θ*_2_ are hyperparameters of this Gaussian kernel. N combinations of sensory evaluation value ***y*** and measured feature vector ***x*** are defined by

$$D=\{\left(x_{1},y_{1}),(x_{2},y_{2}),\ldots ,(x_{N},y_{N}\right)\}.$$ (2)

If a kernel matrix is represented as **K** composed of kernel functions, unknown measured feature vector x* and its predicted sensory value y* are expressed by the following conditional probability of multivariate Gaussian distributions

$$p(y^{*}|x^{*},D)=N(k_{*}^{T}K^{-1}y,k_{**}-k_{*}^{T}K^{-1}k_{*})$$ (3)

where k_*_, k_**_ are expressed by

$$k_{*}={\left(k(x^{*},x_{1}),k(x^{*},x_{2}),\ldots ,(x^{*},x_{N})\right)}^{T}$$ (4)

$$k_{**}=k(x^{*},x^{*})$$ (5)

$k_{*}^{T}K^{-1}y$ in Eq (3) is an expectation value and is also the prediction value of sensory evaluation in this study. The prediction value was calculated by Python 3.10.

In the Gaussian process modeling of each food texture, the mean of the sensory evaluation values and the features of the measured data in the three conditions are used as the dataset as the objective value and the explanatory vector, respectively. The number of datasets was 360. One explanatory vector includes 66 feature values. One GPR model determined the relationship between the objective and explanatory vectors for one texture. In other words, this study made four GPR models. Each GPR model iteratively predicted one sensory evaluation value from one explanatory vector by the leave-one-out cross-validation method. Due to using 360 datasets, the number of iterations of the leave-one-out cross-validation is 360.

## Results and discussion

### Sensory evaluation

**Fig 3:**
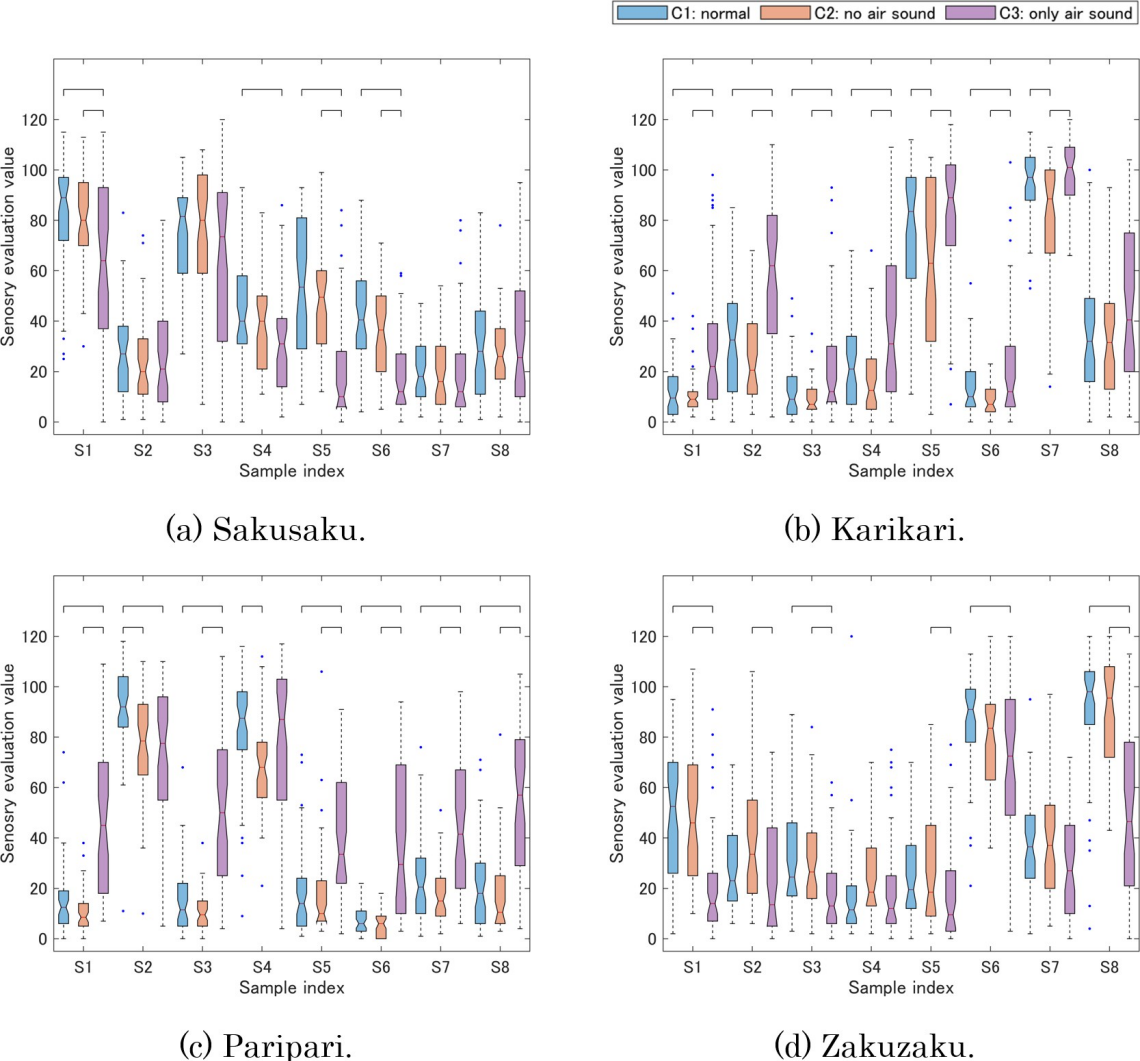
Box plots of four textures in sensory evaluation. The center red line of each box shows median. The upper and lower lines in each box represent the 75th and 25th percentiles, the maximum and minimum values in the whiskers represent the maximum and minimum values for non-outlier data, and the dots represent outliers. Brackets mean the significant difference (p < .05) between the conditions of the same sample. (a) Sakusaku, (b) Karikari, (c) Paripari, and (d) Zakuzaku.

The box plots of sensory evaluation data are shown in Fig 3, which include significant differences (p < .05) between two conditions of a sample calculated by Tukey’s honestly significant difference test.

Regarding conditions 1 (normal) and 2 (no air sound), S5 and S7 of karikari and S2 and S4 of paripari had significant differences, and their medians of condition 1 are higher than those of condition 2 by more than 10. In addition to these cases, the medians in other results tended to be lower due to the absence of sound. Several previous studies have also indicated that air-conduction sound played an important role in crispness [21, 22, 25]. the trend of the present experiment is consistent with these studies.

In comparison between conditions 1 (normal) and 3 (only air sound), more than half of the foods had significant differences in the four textures. Sakusaku and zakuzaku had lower values in condition 3, and this result indicates that sakusaku and zakuzaku are emphasized by force and vibration. On the other hand, karikari and paripari had higher values in condition 3 for almost samples than in condition 1. The differences between the means of the sensory evaluation in conditions 1 and 3 were 11.8 and 19.0 for karikari and paripari, respectively. It is considered that air-conduction sound emphasizes the perception of karikari and paripari.

In comparison between condition 2 (no air sound) and condition 3 (only air sound), although there were some exceptions such as S2 and S4 of Paripari, the relationship between conditions 2 and 3 was mostly like that between conditions 1 and 3. The slight difference between conditions 1 and 2 implied that force and vibration (bone-conduction sound) play a dominant role in the four texture descriptors.

### Instrumental measurement

**Fig 4:**
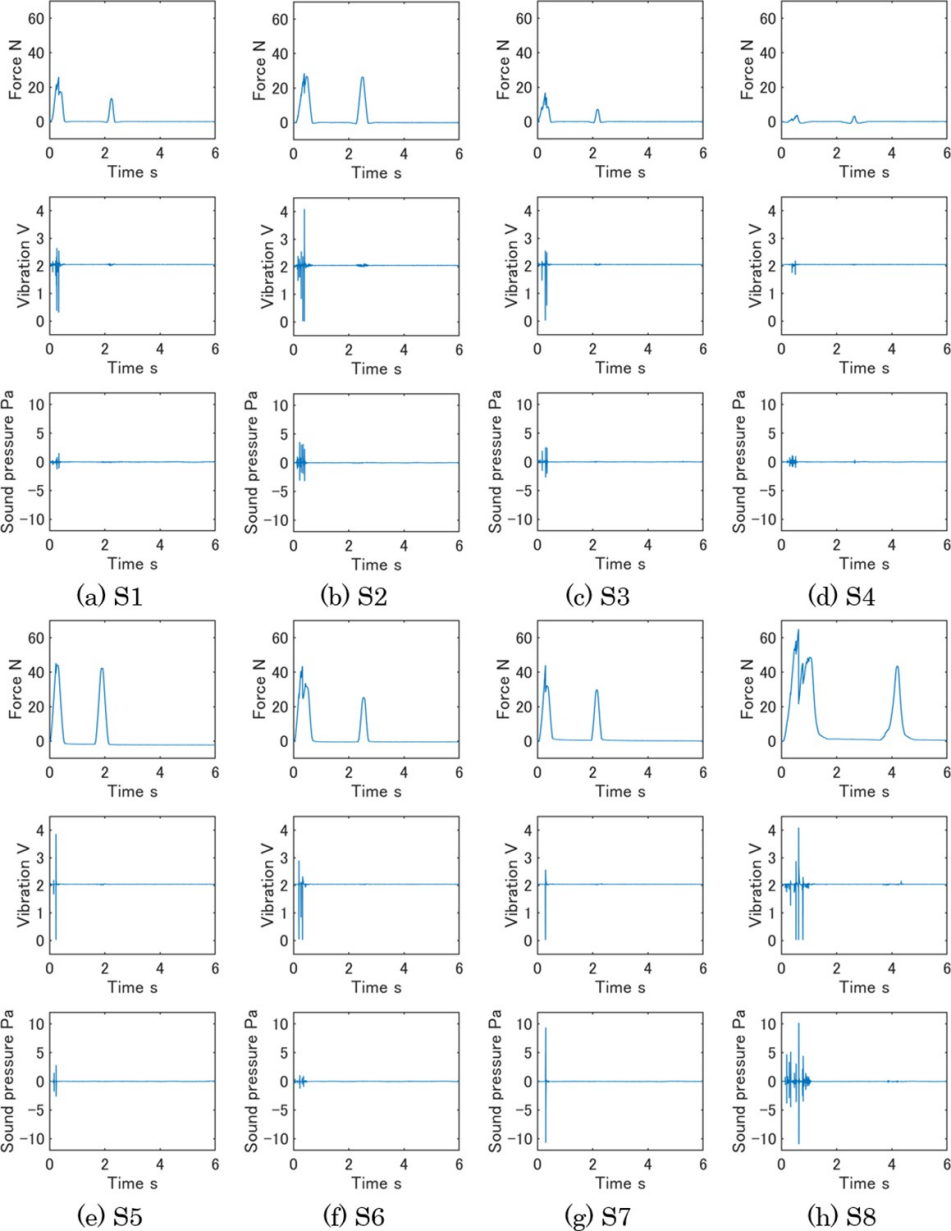
Typical measurement data of eight samples. (a) S1, (b) S2, (c) S3, (d) S4, (e) S5, (f) S6, (g) S7, and (h) S8.

**Fig 5:**
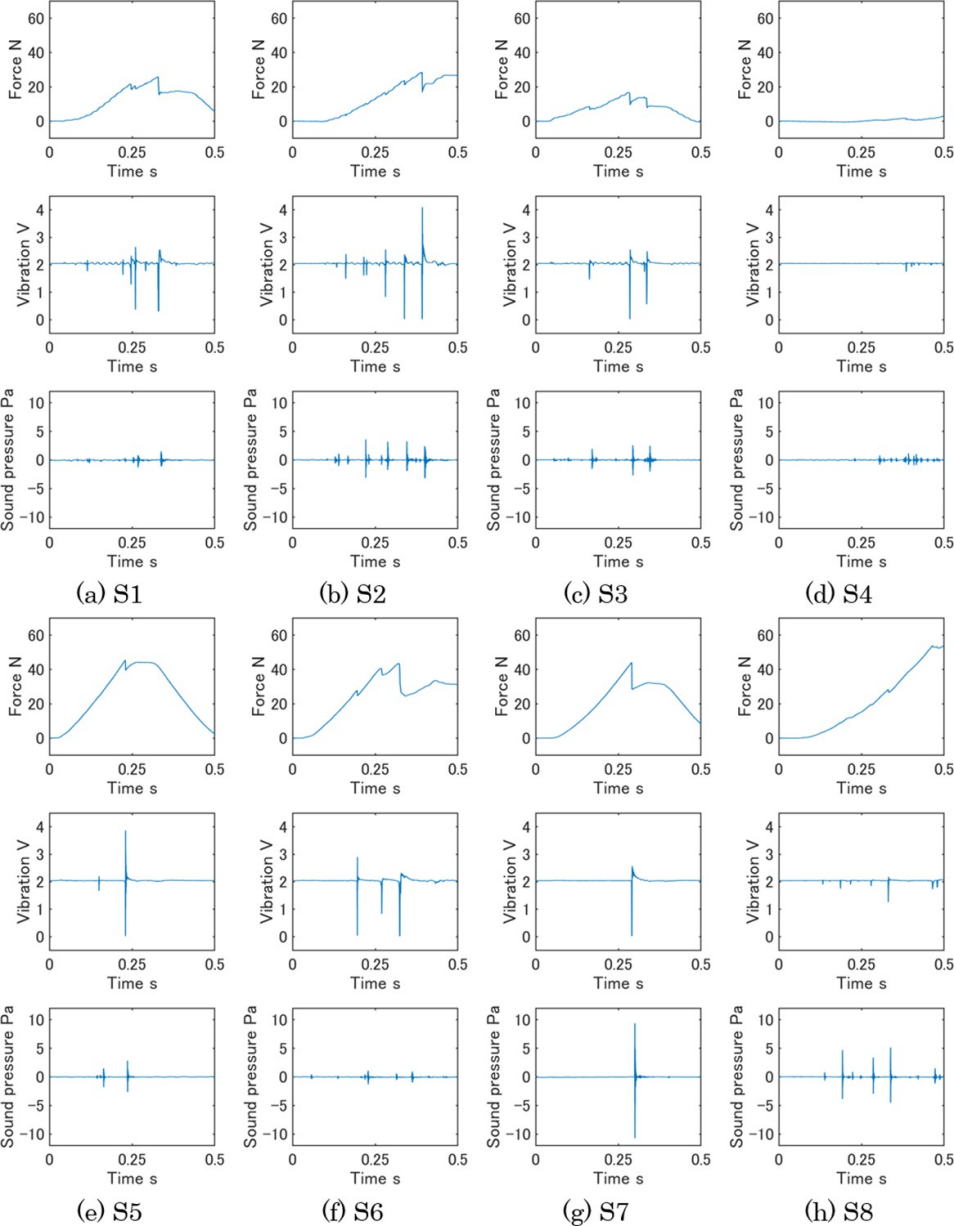
Typical measurement data of eight samples during 0.5 s from the measurement beginning. Each plot corresponds to the plot in Fig 4. (a) S1, (b) S2, (c) S3, (d) S4, (e) S5, (f) S6, (g) S7, and (h) S8.

Typical measurement data under condition 1 are shown in Fig 4. The plots of each sample are arranged in the order of force, vibration, and air-conduction sound from the top to the bottom. Two peaks of force show the data of the first and second compressions and the higher peak shows the harder sample. The unit of the vertical axis in the vibration plots is voltage by electromotive induction. The higher voltage indicates a faster vibration. The vibration and sound data had few peaks at the second compression due to the fracture of samples at the first compression. The vibration and sound data of Fig 4(B) and 4(H) had many spike-shaped peaks, and these samples occurred much vibration to the probe of the sensor and emitted sound. On the other hand, Fig 4(G) included fewer vibration and sound peaks. Fig 5 shows measurement data of eight samples during 0.5 s from the start of measurement. Each plot corresponds to the plot in Fig 4. Fig 5 reveals the detailed change of three measurement data in the first compression.

S1 showed brittleness at the peak of the first compression in force and synchronous high vibrations and small sounds made some peaks. S3 also showed the same trend as S1 in the three data. These two samples had higher evaluation values of sakusaku in the sensory evaluation under condition 1 in Fig 3. So, it is considered these features of the three data were unique for sakusaku. The force of S2 had some peaks before reaching its maximum value, while the vibration and sound had many peaks. S4 was fractured by a low force, and the three data of S4 were low. The measurement data of S2 and S4 seemed different characteristics, however, both S2 and S4 had high values of paripari in sensory evaluation. When the three plots of S2 were compressed in the vertical direction, they were like those of S4. This characteristic, which is the ratio among the three data, may be important for Sakusaku. S5 and S7 fractured at the maximum force, and simultaneously single and high vibrations were measured. Because S5 and S7 had high sensory evaluation values of karikari, the characteristics of these measurement data are unique for karikari texture. S8 had the highest force among the samples. The vibration and sound data showed high peaks. The force and vibration of S6 had similar characteristics to S8. Since S8 and S6 were the samples with high evaluation values of zakuzaku, it is considered that the high peak of force with vibration is a main characteristic of the measurement data of zakuzaku.

### Food texture prediction

**Fig 6:**
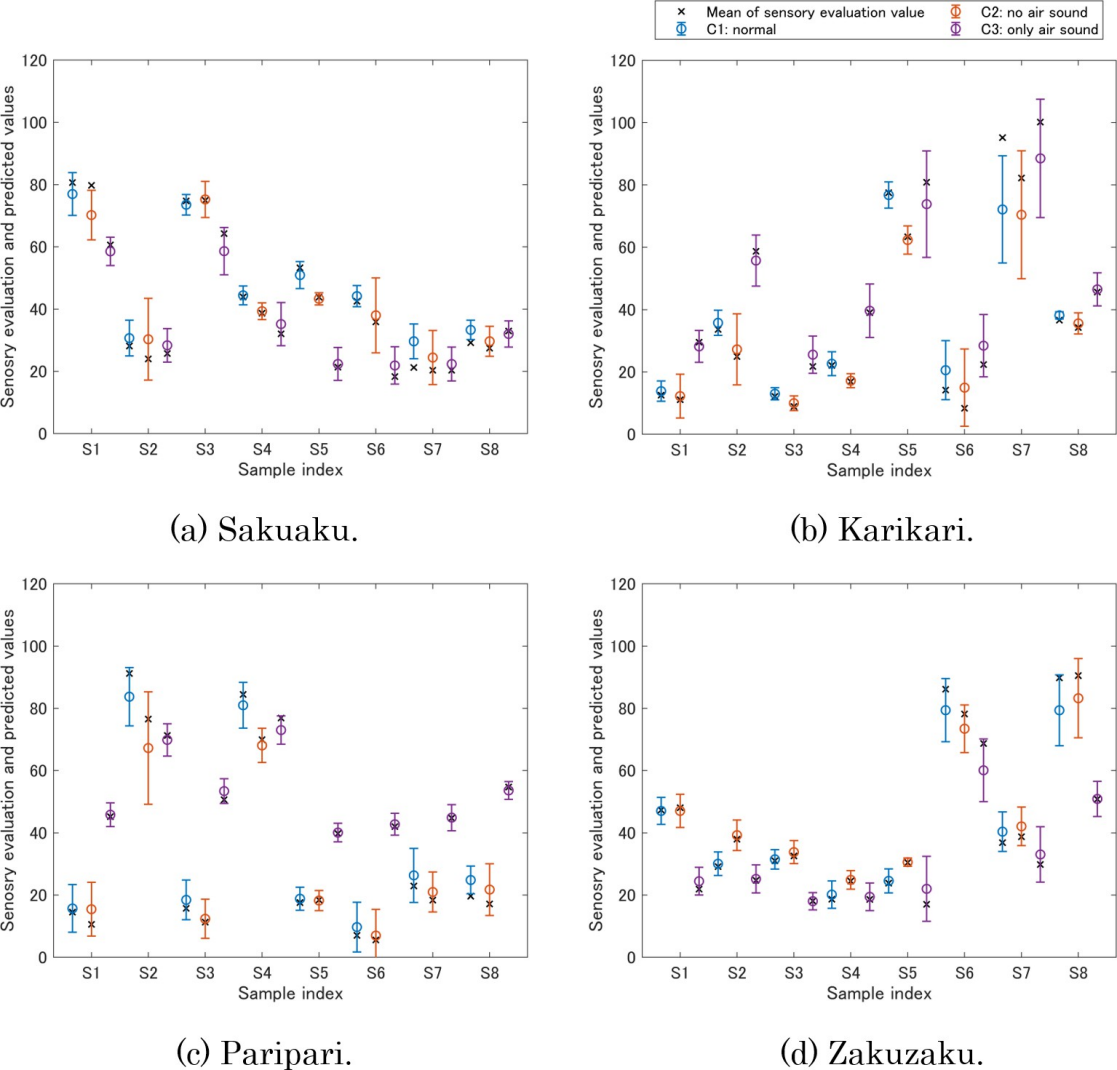
Prediction results of sensory evaluation values. The circles represent the mean of the prediction values, and the error bars represent the range of the standard deviation of the prediction values. The crosses represent the mean of the sensory evaluation values. (a) Sakusaku, (b) Karikari, (c) Paripari, and (d) Zakuzaku.

The mean and standard deviation were calculated from the prediction values of the leave-one-out cross-validation as shown in Fig 6. In many cases in Fig 6, the sensory evaluation values were within the standard deviation of the predicted values, resulting in accurate predictions. In Fig 6(B), the prediction values of S7 had a relatively large difference from the mean value of the sensory evaluation. There are two causes for this result: first, the measurement data of S7 had a variance because of the internal voids in its structure, which induces the data to be variated. The other cause is that the sensory evaluation value of S7 is different from the other samples. The combination of these two causes made accurate prediction of the GPR model difficult. If we improve the latter cause and add samples with a high karikari texture, it is expected that the prediction accuracy will be improved.

Table 4 shows mean absolute errors (MAEs) between the means of the sensory evaluation values and the prediction values. Since the range of the sensory evaluation value was 0–120, an MAE of 6 corresponds to 5% of that range. Except for karikari, the MAEs in Table 4 are less than 6, indicating that the GPR model was able to accurately predict the sensory evaluation values from the instrumental measurement data. In the sensory evaluation results described above, we considered that the sound emphasized paripari. The GPR model for paripari was able to predict this relationship such that paripari was low in conditions 1 and 2, but high in condition 3, as shown in Fig 6(C). Conversely, the sensory evaluation values for sakusaku and zakuzaku in condition 3 were low, but each GPR model was also able to predict their relationships. Hence, it was confirmed that the four food textures including the effects of sensory combinations can be predicted from the instrumental measurement data by the GPR model.

**Table 4:** Mean absolute errors between sensory evaluation values and prediction values of each texture and condition. The bottom row is the mean of each texture.

	Sakusaku	Karikari	Paripari	Zakuzaku
**Condition 1 (normal)**	4.15	6.04	5.97	4.93
**Condition 2 (no air sound)**	6.11	6.71	6.79	4.58
**Condition 3 (only air sound)**	4.64	8.35	3.37	5.69
**Mean**	4.97	7.03	5.38	5.05

The GPR model predicted the sensory evaluation values in conditions 2 and 3 without significant errors. Condition 2 (no air sound) may be effective for the prediction of the texture of elderly people with hard hearing. Condition 3 (only air sound) could be also used to evaluate texture sound on TV or videos on the internet.

This study has limitations. The panels of the sensory evaluation were only young participants about 23 years old. The sensory evaluation should collect participants of a wide range of ages. Under the conditions of this study, the sensations of force and bone-conduction sound were not separated. To investigate the detailed effects of the sensory components, their sensations of them should be separated in sensory evaluation.

## Conclusion

In this study, the effects of the sensory combinations of force, bone-conduction sound, and air-conduction sound on the four food textures was confirmed by sensory evaluation. There was a less significant difference between with or without acoustic sound, but the sensory evaluation values of karikari and paripari were relatively higher in the sound-only condition. The instrumental system also measured force, vibration, and air-conduction sound data under the conditions and the GPR model determined the relationship between the sensory evaluation values and the measurement data. The GPR model predicted the evaluation values from the measurement data. The MAEs between sensory and prediction values were low even with the different sensory combinations. This study confirmed that the four food textures including the effects of sensory combinations can be predicted from the instrumental measurement data by the GPR model.

In the sensory evaluation of this study, the participants were only young people. In the future, the sensory evaluation will be performed on people with other age groups.
